# RNA i-motif landscapes in plant kingdom and their potential functional roles

**DOI:** 10.1093/molbev/msag152

**Published:** 2026-06-20

**Authors:** Bibo Yang, Yuchen Li, Zongyun Yan, Yingying Li, Dilek Guneri, Zoë A E Waller, Yiliang Ding, Huakun Zhang

**Affiliations:** Key Laboratory of Molecular Epigenetics of the Ministry of Education, Northeast Normal University, Changchun, China; Department of Cell and Developmental Biology, John Innes Centre, Norwich Research Park, Norwich, UK; Key Laboratory of Molecular Epigenetics of the Ministry of Education, Northeast Normal University, Changchun, China; Department of Cell and Developmental Biology, John Innes Centre, Norwich Research Park, Norwich, UK; Key Laboratory of Molecular Epigenetics of the Ministry of Education, Northeast Normal University, Changchun, China; School of Health, Science and Society, University of Suffolk, Ipswich, UK; School of Pharmacy, University College London, London, UK; School of Pharmacy, University College London, London, UK; Department of Cell and Developmental Biology, John Innes Centre, Norwich Research Park, Norwich, UK; Key Laboratory of Molecular Epigenetics of the Ministry of Education, Northeast Normal University, Changchun, China

**Keywords:** RNA i-motif, translational regulation, plant evolution, nucleotide composition, environmental adaptation

## Abstract

Nucleotide composition has evolved in patterns that reflect both phylogenetic divergence and environmental adaptation. In plants, our previous analysis of transcriptomes from the 1,000 Plants (1KP) initiative revealed that nucleotide frequencies are significantly associated with habitat temperatures, suggesting selective pressures on RNA sequence and structure. Such pressures may promote the emergence of specific non-canonical RNA motifs, exemplified by G-rich RNA G-quadruplexes, which have been implicated in plant environmental adaptation. Whether similar evolutionary selection operates on other nucleotides and RNA structural motifs, however, remains unclear. Here, we investigate the evolutionary landscape of the RNA i-motif (iM), a C-rich non-canonical RNA structure, across the plant kingdom. Using iM-Seeker, we systematically identified RNA iMs and uncovered a pronounced enrichment within 5′ untranslated regions (5′UTRs), with monocots exhibiting the highest iM abundance among major plant lineages. Integration of ecological variables with iM densities revealed that species from warmer environments preferentially harbor increased numbers of 5′UTR iMs, a trend most evident in monocots. Our translatome analyses in rice, wheat, tomato, and maize further indicate that 5′UTR iMs are generally associated with translational repression. Consistently, experimental validation using orthologous monocot 5′UTRs confirmed that RNA iMs are capable of repressing translation. Together, these findings reveal evolutionary selection on nucleotide composition and RNA structural motifs, highlight the adaptive significance of RNA i-motifs, and suggest that plants may use i-motifs as molecular signatures to facilitate environmental adaptation during evolution.

## Introduction

Earth’s remarkable biological diversity has emerged largely through the continual adaptation of organisms to distinct ecological niches, a process that underlies lineage divergence and speciation (Šmarda et al. 2014; Leebens-Mack et al. 2019; Hou et al. 2025). Plants provide a striking example of this evolutionary principle (Kang et al. 2023). As sessile organisms, they must endure and respond to fluctuating local environments, a constraint that over evolutionary time has driven the emergence of highly specialized physiological and molecular traits (Leebens-Mack et al. 2019; Yang et al. 2022). This adaptive diversification has enabled plants to colonize an exceptional range of habitats across the globe (Savolainen et al. 2013).

**Figure 1:**
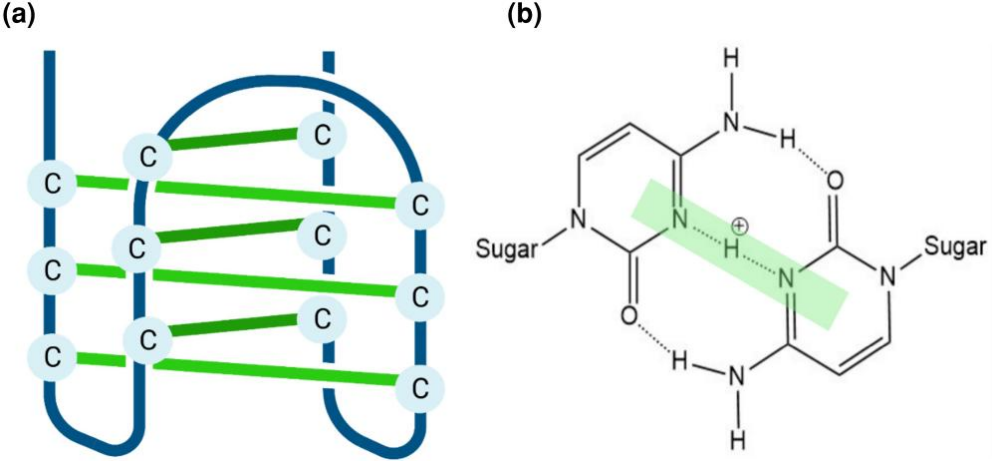
The schematic of i-motif. (a) The structure of i-motif. (b) The demonstration of hemi-protonated C-neutral C base pairs. Created with BioRender.com.

At the molecular level, the four nucleotide bases of RNA—adenine (A), uracil (U), cytosine (C), and guanine (G)—give rise to a wide array of sequence and structural configurations, enabling diverse biological functions (Yang et al. 2022). Variations in genomic nucleotide composition have been proposed to contribute to the ability of plant species to adapt to and ultimately thrive within specific environmental niches (Šmarda et al. 2014; Yang et al. 2022). Our previous systematic transcriptome-wide investigation across the plant kingdom revealed distinct differences in nucleotide composition across both different species and different genic regions (Yang et al. 2022). Notably, correlations between nucleotide density and climatic variables revealed significant associations with habitat temperature (Yang et al. 2022). Among these, guanine exhibited a strong negative correlation with temperature-related bioclimatic variables, indicating an enrichment of G in species adapted to colder environments. This pattern is supported by the properties of RNA G-quadruplexes (GQSs), G-rich non-canonical RNA structures that display enhanced stability at low temperatures and can protect transcripts from degradation (Yang et al. 2022). Such findings provide a molecular rationale for the selective enrichment of G in cold-adapted plant species. Intriguingly, we also observed a distinct pattern for cytosine. Across most plant species, cytosine is the least abundant nucleotide within coding sequences (CDS) and 3′ untranslated regions (3′UTRs), yet its frequency increases markedly within 5′ untranslated regions (5′UTRs) (Yang et al. 2022). In many dicots, C exceeds G in abundance in 5′UTRs, and in numerous monocots, it becomes the most prevalent nucleotide (Yang et al. 2022). These observations suggest that cytosine enrichment in 5′UTRs may have evolved to support specialized regulatory functions. Cytosine-rich sequences are capable of forming iMs, the four-stranded non-canonical DNA and RNA structures stabilized by intercalated C-rich tracts through hemi-protonated C·C^+^ base pairing (Fig. 1) (Abou Assi et al. 2018). Genome-wide analyses have shown that iMs are enriched in key regulatory regions, including promoters, telomeres, and transposable elements, implicating them in diverse biological processes such as transcriptional regulation, DNA replication, transposon activity, and cell-cycle control (Abou Assi et al. 2018; Zeraati et al. 2018; Tao et al. 2024). Although biophysical studies have confirmed the formation of individual RNA i-motif (iM) structures (Snoussi et al. 2001; Preciado et al. 2026), the prevalence, distribution, and functional significance of RNA iMs at the transcriptome scale remain largely unexplored.

Plants provide an ideal system for investigating the landscape and functional significance of RNA iMs, owing to their exceptional diversity and adaptation to distinct ecological habitats. Here, we systematically identified the RNA iM landscapes across diverse plant transcriptomes using our iM-Seeker framework (Yang et al. 2024), generating the first transcriptome-wide iM atlases across the plant kingdom. iM-Seeker is the first prediction tool developed based on iM–specific experimental data. Within this framework, it enables both the identification of potential iM–forming sequences and the assessment of their folding potential, making it a reliable and comprehensive approach for iM prediction (Yang et al. 2024). We observed a pronounced enrichment of iMs within the 5′UTR regions across plant species, with a particularly high frequency in monocots. By integrating 19 bioclimatic variables corresponding to species’ habitats from the Global Biodiversity Information Facility (GBIF), we further found that plants growing in warmer environments tend to retain substantially higher numbers of 5′UTR iMs. We next analysed the translatomes in rice, wheat, tomato, and maize and revealed that 5′UTR iMs are associated with translational repression. Our further experimental validation using orthologous monocot 5′UTRs confirmed that RNA iMs are capable of repressing translation. In summary, our results position RNA iMs as evolutionarily selected structural signatures that associate nucleotide composition with environmental adaptation, revealing an additional layer by which plants tune gene regulation in response to ecological pressures.

## Results

### The transcriptome-wide iM atlases in plant kingdom

**Figure 2:**
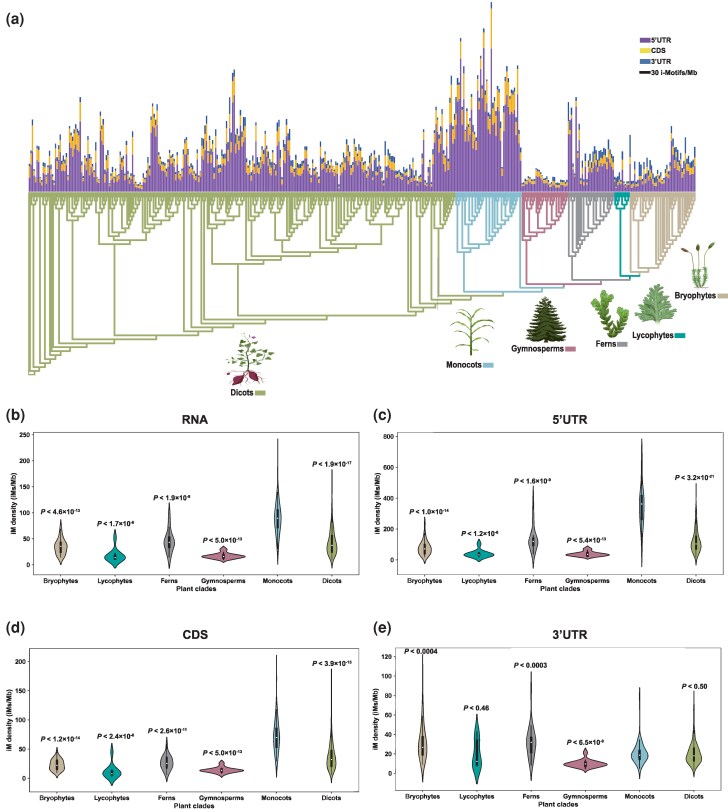
The transcriptome-wide iM atlases in plant kingdom. (a) The iM densities (iMs/Mb) of various genic regions (5′UTR, CDS, 3′UTR) across 433 land plants. The plants are in six clades. N = 277, 43, 30, 30, 10, 43 for dicots, monocots, gymnosperms, ferns, lycophytes, and bryophytes, respectively. The distribution of iM density in genic regions across the whole transcriptome (b), 5′UTR s (c), CDSs (d), 3′UTR s (e). Statistical analysis was performed between monocots and the other five plant clades, with significance tested by the Mann-Whitney *U* test.

Building on our previous work (Yang et al. 2022), we integrated transcriptomes from the 1,000 plants initiative, namely 1KP (Leebens-Mack et al. 2019), with habitat locations obtained from the GBIF. This dataset comprised 433 plant species spanning six plant clades, including dicots (N = 277), monocots (N = 43), gymnosperms (N = 30), ferns (N = 30), lycophytes (N = 10), and bryophytes (N = 43) (Yang et al. 2022). Using the iM-Seeker framework (Yang et al. 2024), we systematically identified iMs across all transcriptomes. To avoid redundancy, iMs were counted using the “non-overlapped” and “non-greedy” configurations. Identified iMs were classified according to genic regions (5′UTR, CDS, and 3′UTR). iM density, defined as the number of iMs per megabase, was used as a measure of iM abundance (Mullen et al. 2010). We calculated iM densities for each genic region across all plant species (Figure S1). Among the three regions, 5′UTRs consistently exhibited significantly higher iM densities than CDSs and 3′UTRs across all clades (5′UTR vs CDS *P* < 10^−74^, 5′UTR vs 3′UTR *P* < 10^−71^, Figure S1). Notably, monocots displayed overall higher iM densities in both 5′UTR and CDS regions compared with the other five plant clades (Fig. 2), suggesting a broader adoption of iMs across monocot transcriptomes. To account for potential bias arising from differences in cytosine (C) content among species, we further calculated C density (percentage of cytosine among the four nucleotides) and defined iM enrichment as the number of iMs per megabase of cytosine. We then compared both iM density and iM enrichment to assess whether the observed differences in iM density could be attributed to variation in C content. Although significant differences in C density were observed across genic regions between monocots and species from the other five clades (Figure S2), the differences in iM enrichment were substantially pronounced (Figure S3). For example, the marked divergence between monocots and dicots in 5′UTRs was evident at both the iM density level (*P* < 3.2 × 10^−21^) and iM enrichment level (*P* < 1.9 × 10^−19^), indicating that cytosine content alone cannot explain the elevated iM abundance in monocots (Fig. 2c; Figure S3b). Together, these results underscore the enrichment of iMs in 5′UTRs and their widespread prevalence—particularly in monocots—across plant transcriptomes.

To further characterize the diversity of iMs across the plant kingdom, we classified iMs into five categories based on the number of C-tract layers and the length of the longest loop. Type 1 iMs contain three-layer C-tracts with the longest loops of 1 to 4 nucleotides; Type 2 iMs contain three-layer C-tracts with the longest loops of 5 to 8 nucleotides; Type 3 iMs contain three-layer C-tracts with loops of 9 to 12 nucleotides; Type 4 iMs contain four-layer C-tracts; and Type 5 iMs contain more than four layers of C-tracts. We quantified the distribution of these iM types across 433 plant species and found that Types 2 and 3 predominated across all clades (Table S1; Figure S4). Collectively, the five types accounted for 11.67%, 29.29%, 55.84%, 2.98%, and 0.22% of all iMs, respectively, with the majority (85.13%) corresponding to three-layer iMs with the longest loop lengths of 5 to 12 nucleotides (Table S1). Notably, ferns exhibited the highest proportion of iMs containing more than three C-tract layers (4.95%), substantially exceeding that observed in lycophytes (3.81%), which ranked second among the clades. To further assess iM properties, we used iM-Seeker to estimate the folding strength (stability) of individual iMs across the six plant clades (Yang et al. 2024). Consistent with their higher proportion of multi-layer iMs, ferns were predicted to form relatively more stable iMs than other clades, suggesting a greater reliance on stable iMs for functional roles (Figure S5). Taken together, these results reveal a conserved dominance of three-layer iMs with intermediate loop lengths across plant transcriptomes, while highlighting lineage-specific enrichment of structurally complex and more stable iMs in ferns, suggesting evolutionary diversification in iM architectures among plant clades.

### Transcriptomic iM abundances in 5′UTRs exhibit climatic adaptation

**Figure 3:**
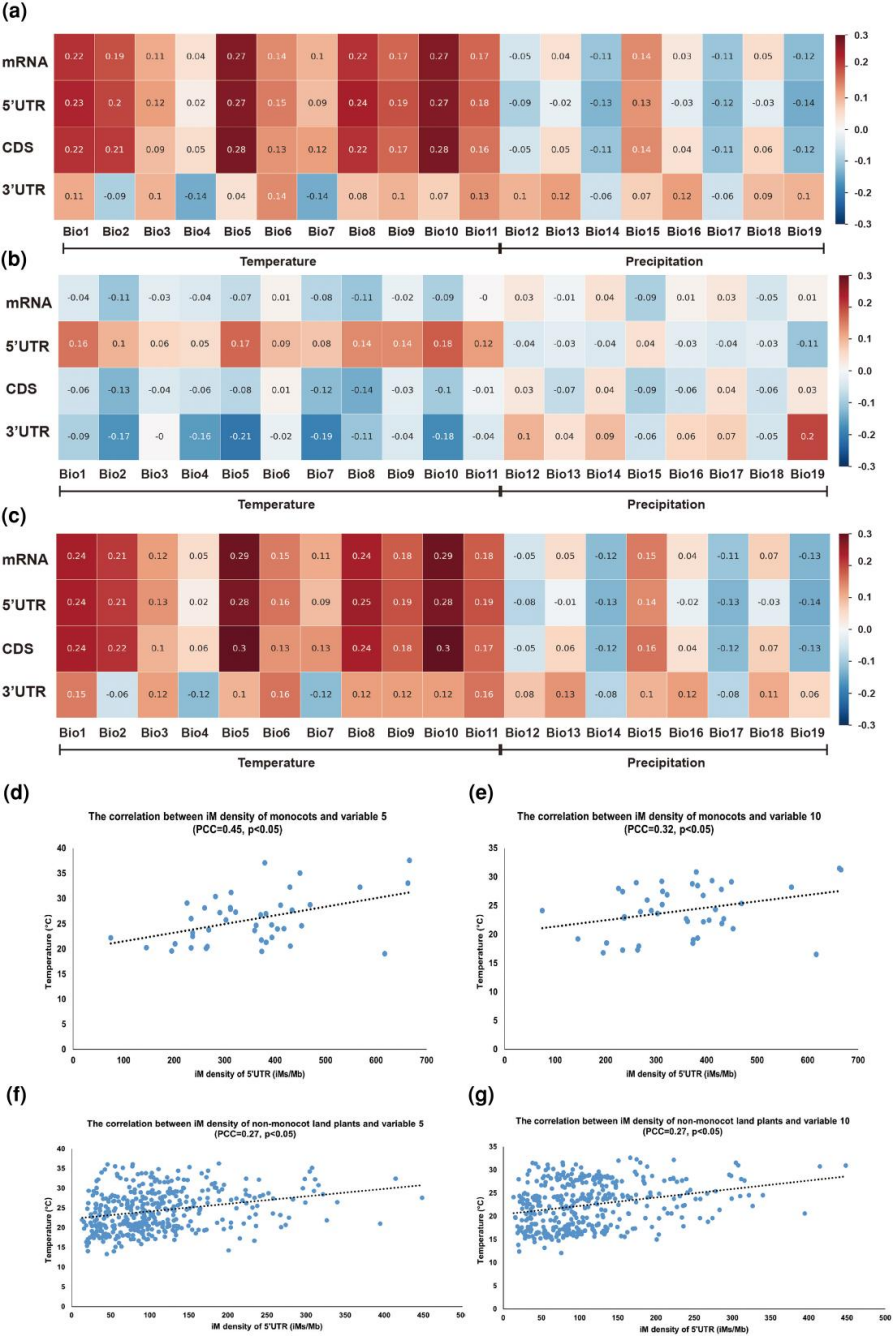
The associations between iM abundances and environmental variables. (a) The Pearson Correlation Coefficients (PCCs) between iM density and associated bioclimatic variables related to the habitats of 433 land plants. (b) The heat plot showing the PCCs between cytosine density and associated bioclimatic variables related to the habitats of 433 land plants. (c) The heat plot showing the PCCs between iM enrichment (iMs/Mb cytosines) and associated bioclimatic variables related to the habitats of 433 land plants. (d) The PCCs between iM density and temperature-significant variables BIO5 (max temperature of the warmest month) across monocot land plants. (e) The PCCs between iM density and temperature-significant variables BIO10 (mean temperature of the warmest quarter) across monocot land plants. The PCCs between iM density and two temperature-significant variables BIO5 (f) and BIO10 (g) (BIO5: max temperature of the warmest month; BIO10: mean temperature of the warmest quarter) across non-monocot land plants.

Next, we measured the relationships between iM density and bioclimatic variables by calculating Pearson correlation coefficients (PCCs) (Fig. 3). Strongly significant positive correlations between iM density and temperature-related variables were observed in both 5′UTR and CDS regions (Fig. 3a), whereas correlations with precipitation-related variables were generally weakly positive or negative (Fig. 3a). To disentangle the contribution of nucleotide composition, we further calculated PCCs between cytosine (C) density and bioclimatic variables, as well as between iM enrichment (iMs per megabase cytosine) and bioclimatic variables (Fig. 3b and c). Overall, C density exhibited weakly positive or negative correlations with temperature variables, whereas iM enrichment showed consistently stronger positive correlations, indicating that temperature-associated iM patterns cannot be explained solely by cytosine content (Fig. 3b and c). Among the eleven temperature-related variables, BIO5 (maximum temperature of the warmest month) and BIO10 (mean temperature of the warmest quarter) showed the strongest correlations with iM density in 5′UTRs (BIO5 = 0.27; BIO10 = 0.27; Fig. 3a). We therefore examined these relationships separately in monocots and in the remaining plant species (Fig. 3d–g). Notably, monocots exhibited substantially higher PCCs than other plants (monocots: BIO5 = 0.45, BIO10 = 0.32; other plants: BIO5 = 0.27, BIO10 = 0.27), suggesting a stronger association between 5′UTR iM density and temperature in monocots. In order to avoid the correlation bias caused by phylogenetic dependence across species, we also performed phylogenetic generalized least squares (PGLS) analyses to re-evaluate the relationships between environmental variables and iM density, iM enrichment, and C density. PGLS analyses were performed using a Brownian motion model of trait evolution, with the phylogenetic covariance structure specified using the species tree. The results are summarized in Tables S2–S4 and remain highly consistent with our original PCC findings. For example, PGLS coefficients of both 5′UTR iM density and iM enrichment show predominantly positive correlations with temperature-related environmental variables. Consistently, their PGLS coefficients for BIO5 and BIO10 are significantly positive and of large magnitude.

Consistent with this observation, monocots inhabit significantly warmer environments than other plant species, as reflected by higher mean values of both BIO5 (monocots: 25.86 °C; other plants: 24.35 °C) and BIO10 (monocots: 24.17 °C; other plants: 22.43 °C) (Figure S6). Collectively, these results indicate that plants adapted to warmer climates tend to harbor higher iM abundance in their 5′UTRs, a trend that is particularly pronounced in monocots. This temperature-associated enrichment of iMs in monocots may represent an evolutionary molecular signature reflecting adaptation to warm habitats.

### i-motifs in plant 5′UTRs are associated with repressed translation efficiency

Given the pronounced enrichment of iMs in plant 5′UTRs, we next investigated their potential functional roles in translational regulation. Previous studies have demonstrated that RNA structures within 5′UTRs can strongly influence translation by impeding ribosome loading, altering pre-initiation complex scanning, and affecting start codon selection (Cao et al. 2024; Zhang and Ding 2025). Known translation-regulatory structures include hairpin motifs and RNA GQSs, another class of non-canonical RNA structures (Yang et al. 2020, 2021). These findings prompted us to hypothesize that iMs in 5′UTRs may similarly modulate translation.

**Figure 4:**
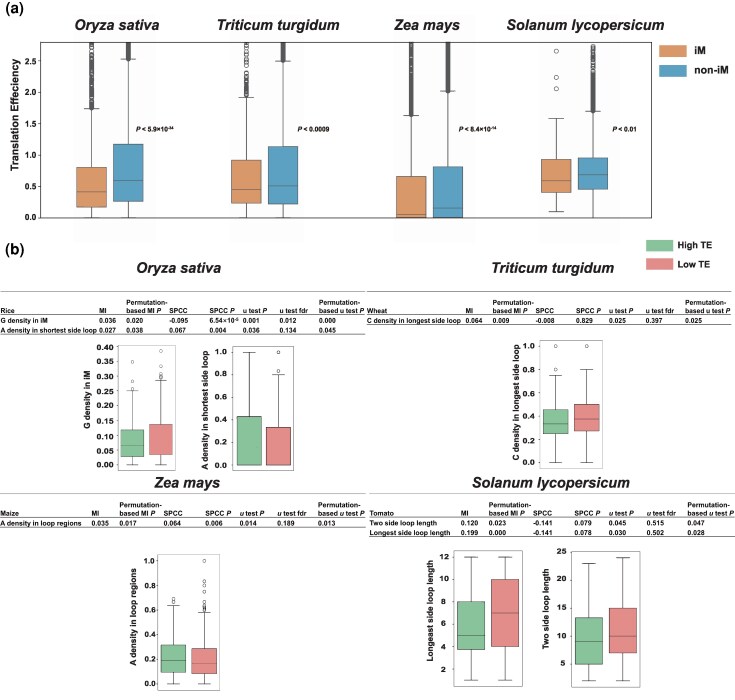
i-Motifs in 5′UTRs are associated with repressed translation efficiency. (a) The distribution of translation efficiencies across four species: rice, durum wheat, tomato, and maize. Statistical analysis of TE between two groups was performed, with significance tested by the Kolmogorov-Smirnov test. (b) The association between TE and iM features of four species. The statistic measures included MI, permutation-based MI significance test, Spearman correlation coefficient (SPCC) and significance, Mann–Whitney U test, *U* test after Benjamini–Hochberg (FDR) correction, and permutation-based significance test on *U* test. The important features were selected according to three criteria (permutation-based MI *P* < 0.05; *P* value of Mann-Whitney *U* test < 0.05; permutation-based *U* test *P* < 0.05). Box plots illustrate the distributions of the high-TE group and the low-TE group.

To test this hypothesis, we collected published ribosome profiling data for tomato (*Solanum lycopersicum*) and maize (*Zea mays*), as well as polysome profiling data for rice (*Oryza sativa* L. ssp. *japonica*) and Kronos wheat (*Triticum turgidum* ssp. *durum*). We reanalyzed these datasets by mapping raw reads to their respective transcriptomes, quantifying expression levels as FPKM, and calculating translation efficiency (TE). We then compared TEs between genes with and without iMs in their 5′UTRs (Fig. 4a). Across all four species, genes harboring iMs in their 5′UTRs exhibited significantly lower TEs than genes lacking iMs, indicating that 5′UTR iMs are associated with translational repression (Fig. 4a).

To identify iM-specific features underlying translational suppression, we extracted 34 features from individual 5′UTR iMs in the four species, including 9 length-related features, 24 nucleotide density features, and predicted iM folding strength (stability). We assessed the relationships between these features and TE using mutual information (MI), Spearman correlation coefficients (SPCCs), and Mann–Whitney *U* tests comparing genes with high versus low TE. Features with permutation-based MI *P* < 0.05, *P* value of Mann-Whitney *U* test < 0.05, and permutation-based *U* test *P* < 0.05 were considered informative and are summarized in Fig. 4b. Our analyses revealed that both nucleotide composition and loop length are key determinants of iM-mediated translational regulation. In particular, adenine (A), guanine (G), and cytosine (C) densities within loop regions emerged as the most influential features, consistent with previous studies showing that loop nucleotide composition plays a critical role in stabilizing iM structures (Fojtík and Vorlícková 2001; Wright et al. 2017). Our results suggested that certain iMs with different A, G, and C densities in loops may affect their regulation of translation.

### Experimental validation of i-motif–mediated translational repression

**Figure 5:**
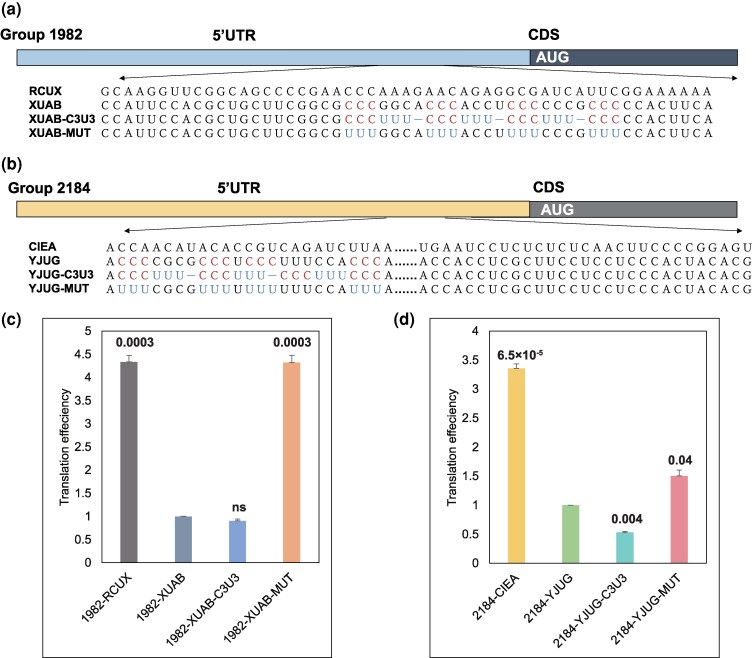
Experimental validation of i-Motif–mediated translational repression. The design of the dual luciferase reporter of orthologous groups 1982 (a) and 2184 (b). The C tracts of putative iMs are highlighted in red, while the mutated nucleotides are blue. The abbreviations of the species are as follows: *Maianthemum sp.* (RCUX), *Paraneurachne muelleri* (XUAB), *Juncus inflexus* (CIEA), and *Curculigo sp.* (YJUG). The comparison of translation efficiency (TE) of groups 1982 (c) and 2184 (d) was measured by dual luciferase reporter assay. Statistical analysis of translation efficiency between 1982-XUAB and other candidates in group 1982 and 2184-YJUG and other candidates in group 2184 was performed by Student’s *t*-test with four biological replicates. Data are presented as mean values + standard error of mean (SEM).

To validate the trends observed in our meta-analysis, we performed experimental assays to directly assess the impact of putative iMs on translation. We focused on monocot species and selected two orthologous gene groups (IDs 1982 and 2184) as representative examples from pre-identified orthologous gene groups in the 1,000 Plants initiative (1KP) (Leebens-Mack et al. 2019). From each group, we analyzed two orthologous transcript variants: one harboring an iM within the 5′ untranslated region (5′UTR) and a corresponding variant lacking the iM sequence. For each iM-containing candidate, we designed two types of mutations. The first converted the native iM sequence into a (C_3_U_3_)_3_C_3_ variant, which has been reported to preferentially fold (Preciado et al. 2026). The second mutation replaced all cytosines in the C-tracts with uracils, thereby abolishing the iM structure (Fig. 5a and b). We found that transcripts containing iM-forming sequences exhibited significantly lower translation efficiencies (TEs) than their non-iM counterparts in two selected candidate orthologous gene groups (*Maianthemum sp.* RCUX vs *Paraneurachne muelleri* XUAB, *P* = 3 × 10^−4^; *Juncus inflexus* CIEA vs *Curculigo sp.* YJUG, *P* = 6.5 × 10^−5^). Notably, the (C_3_U_3_)_3_C_3_ mutation markedly reduced translational efficiency, almost to the level of native transcripts with iM in 5′UTRs, suggesting that iMs suppress translation in general. In contrast, disruption of the iM through cytosine-to-uracil substitution led to a pronounced and significant increase in TE in both groups (XUAB-MUT > XUAB, *P* = 3 × 10^−4^; YJUG-MUT > YJUG, *P* = 0.04), indicating that disruption of iMs relieves translational suppression. Taken together, these results provide direct experimental evidence that iMs in plant 5′UTRs act as translational repressors, likely by impeding the translation machinery.

## Discussion

### Evolutionary selection of iMs enrichment in 5′UTRs

Nucleotide composition is not a neutral property of transcriptomes but rather reflects a complex interplay between phylogenetic history, environmental pressures, and functional constraints (Šmarda et al. 2014). In the plant kingdom, C is the least abundant nucleotide in CDS and 3′UTR, yet in the 5′UTR, its relative abundance increases, even surpassing G in dicots and becoming the most frequent nucleotide in many monocots (Yang et al. 2022). One of the most striking observations from our analysis is the pronounced enrichment of iMs within 5′UTRs across the plant kingdom (Figure S1; Fig. 2). This may help explain the cytosine enrichment in plant 5′UTRs. Notably, we also calculated the iM densities across different genic regions in both human and mouse transcriptomes. In both species, the iM densities in the 5′UTR are substantially higher than those in the CDS and 3′UTR. For example, in humans, the iM density reaches 807.74 iM/Mb in the 5′UTR, compared to 272.37 iM/Mb in the CDS and 319.54 iM/Mb in the 3′UTR. A similar pattern is observed in the mouse, with iM densities of 573.31 iM/Mb in the 5′UTR, 186.59 iM/Mb in the CDS, and 217.06 iM/Mb in the 3′UTR. These results suggest that the enrichment of iMs in 5′UTRs is unlikely to be plant-specific and may instead represent a conserved feature across eukaryotic transcriptomes. Importantly, our normalization of iM abundance by cytosine content demonstrates that iM enrichment is not a trivial consequence of nucleotide composition alone. Instead, these data indicate an intrinsic preference for iM formation in 5′UTRs, suggesting that selective pressures act specifically on RNA structural potential rather than merely on sequence composition.

### Climatic adaptation and temperature-associated iM enrichment

Our integration of bioclimatic data reveals a robust association between iM abundance—particularly in 5′UTRs—and environmental temperature. Species inhabiting warmer environments consistently harbor higher densities of 5′UTR iMs, a pattern most pronounced in monocots (Figs. 2 and 3), which themselves are distributed predominantly in warmer climates. This temperature-associated enrichment mirrors, in an opposing direction, the previously reported enrichment of G-rich RNA GQSs in cold-adapted plant species (Yang et al. 2022). Together, these findings suggest a broader evolutionary principle: distinct non-canonical RNA structures may be selectively favored under different thermal regimes.

Beyond their distribution, iMs also exhibit lineage-specific structural features. Ferns, for example, display a higher proportion of iMs with longer C-tracts and greater predicted folding stability, as well as a notable enrichment of iMs in 3′UTRs (Fig. 2; Figure S5). This pattern parallels the established role of stable 3′UTR GQSs in transcript stabilization and suggests that iMs in ferns may have diversified to fulfill distinct post-transcriptional regulatory functions. Such variation underscores the evolutionary plasticity of iM architecture and suggests that different plant lineages may exploit iMs in context-dependent ways.

### iMs as translational regulatory elements

Our translatome analyses and experimental validations collectively demonstrate that iMs in 5′UTRs are generally associated with translational repression (Figs. 4a and 5). This observation is consistent with a well-established paradigm in which stable RNA structures in 5′UTRs impede ribosome scanning and pre-initiation complex progression (Yang et al. 2021; Cao et al. 2024; Zhang and Ding 2025). Importantly, our findings place iMs alongside hairpins and RNA GQSs as functionally relevant RNA structures capable of modulating translation.

Notably, translational repression did not strongly correlate with predicted iM folding strength, suggesting that static structural stability alone does not determine regulatory impact (Fig. 4b). Instead, feature-level analyses point to loop nucleotide composition—particularly adenine, cytosine, and guanine content—as a key determinant of translation regulation (Fig. 4b). The nucleotide composition of iM loops may play an important role in modulating translational repression through several, non-mutually exclusive mechanisms. First, loop sequences can influence the stability and folding dynamics of the iM structure (Wright et al. 2017; Abou Assi et al. 2018; Tao et al. 2024). Differences in nucleotide composition may alter loop flexibility and intramolecular interactions, thereby affecting overall iM stability (Tao et al. 2024). For instance, A and G residues in loops are known to destabilize iMs (Wright et al. 2017; Tao et al. 2024; Yang et al. 2024). More stable iMs within the 5′UTR could more effectively impede ribosome scanning or initiation complex assembly, resulting in stronger translational repression. Second, loop regions may act as binding platforms for RNA-binding proteins (RBPs). Specific nucleotide compositions could promote or restrict the recruitment of regulatory factors that stabilize the iM structure or directly repress translation, adding an additional layer of regulatory specificity beyond the core C-rich tracts. Third, loop composition may influence the structural interplay between iMs and other RNA secondary structures within the 5′UTR. Certain loop sequences could favor or disrupt alternative conformations, thereby modulating ribosome accessibility or the formation of competing regulatory elements.

The enrichment of iMs observed in warm-adapted plants may have important implications for understanding and engineering climate resilience in crops. One possibility is that iM structures, particularly those located in 5′UTRs, function as temperature-responsive regulatory elements that modulate translation efficiency. If iM formation or stability is sensitive to temperature, these structures could act as molecular switches to fine-tune protein synthesis under heat stress conditions. This raises the potential for exploiting iM-forming sequences in crop genetic engineering. For instance, incorporating or optimizing iM elements within the 5′UTRs of stress-responsive genes could enable more precise translational control under elevated temperatures, thereby enhancing stress tolerance without requiring constitutive overexpression. Moreover, comparative analyses between warm-adapted and less heat-tolerant species may help identify sequence features—such as cytosine tract length or loop composition—that contribute to iM stability and functionality under high-temperature conditions. These features could inform the rational design of synthetic regulatory elements for improved gene expression control in crops. Although these possibilities remain to be experimentally validated, they suggest that iM–mediated regulation may represent a promising and largely unexplored avenue for developing crops with enhanced resilience to climate change.

Taken together, our findings position RNA iMs as evolutionarily selected, environmentally responsive regulatory elements that contribute to the regulation of gene expression in plants. By integrating nucleotide composition, RNA structure, environmental temperature, and translational regulation, our work provides a framework for understanding how RNA structure may participate in adaptive regulatory processes.

More broadly, our results suggest that non-canonical RNA structures may serve as molecular archives of evolutionary history, encoding information about environmental pressures within transcriptomes. As such, RNA iMs represent not only functional regulatory elements but also evolutionary signatures of plant adaptation to diverse ecological niches.

## Conclusion

In conclusion, we provide the first comprehensive transcriptome-wide analysis of RNA iMs across the plant kingdom and demonstrate their evolutionary, environmental, and functional relevance. RNA iMs are strongly enriched in 5′UTRs across plants, with particularly high abundance in monocots, and this enrichment is largely independent of cytosine content. Integration with ecological data reveals that 5′UTR iM abundance positively correlates with environmental temperature, suggesting selective retention of iMs in warm-adapted species. Functional analyses further show that 5′UTR iMs are associated with translational repression, supported by both translatome datasets and experimental reporter assays. Together, our results establish RNA iMs as conserved, environmentally responsive regulatory elements that link nucleotide composition, RNA structure, and gene regulation during plant adaptation.

## Materials and methods

### iM identification and analysis of environmental variables

To explore the iM landscapes across the plant kingdom and their associations with the corresponding habitat environments, we integrated transcriptomes from the 1,000 Plants initiative (1KP) (Leebens-Mack et al. 2019) with habitat locations obtained from the GBIF. This dataset comprised 433 plant species spanning six plant clades (Yang et al. 2022). The geographic distribution information (latitude and longitude of representative habitats) of 433 land plants was collected from the GBIF (www.gbif.org). According to the obtained latitude and longitude, nineteen environmental variables of 433 species with over 100 observations were collected from the WorldClim database (Fick and Hijmans 2017; Yang et al. 2022). For each variable per plant species, the 10th, 25th, 50th, 75th, and 90th quartiles were calculated to summarize the climatic distribution for each species. The details of these environmental variables are shown as follows:

BIO1: Annual mean temperature
BIO2: Mean diurnal temperature range (monthly mean of max temp − min temp)
BIO3: Isothermality (BIO2/BIO7 × 100)
BIO4: Temperature seasonality (standard deviation × 100)
BIO5: Maximum temperature of the warmest month
BIO6: Minimum temperature of the coldest month
BIO7: Annual temperature range (BIO5 − BIO6)
BIO8–BIO11: Mean temperatures of the wettest, driest, warmest, and coldest quarters, respectively
BIO12–BIO19: precipitation variables in different conditions, including annual, wettest month, driest month, seasonality (coefficient of variation), wettest quarter, driest quarter, warmest quarter, and coldest quarter.
The transcriptome reference sequences of human (GRCh38.p14) and mouse (GRCm39) were obtained from the Ensembl database.

Our iM-Seeker (command-line version) was first applied to search putative iMs on all collected transcriptomes using a non-overlapping and non-greedy strategy “–overlapped 2 –greedy 2 –representative_conformation 1” (Yang et al. 2024). Only iMs with “+” as the last letter of the iM ID were selected. The search for iMs on the complementary sequences is only applied to DNAs, not RNAs. The Pearson Correlation Coefficient (PCC) and significance between iM densities and bioclimatic variables were performed by in-house Python scripts, and the corresponding *P* values were adjusted by the Benjamini–Hochberg method (FDR). The lowest FDR among the different quartiles of climate features was used to represent the corresponding variables following our previous study (Yang et al. 2022). The Pearson correlation coefficient (PCC) analyses between bioclimatic variables and both iM enrichment and C density were performed using the same quartile data as used for iM density. The PGLS analyses were performed using “ape” and “nlme” packages in the R language (Pinheiro et al. 2017; Paradis and Schliep 2019).

### Calculation of translation efficiency

We collected polysome-seq of rice (BioProject ID number PRJNA1112739) and wheat (BioProject ID number PRJNA723219) data and ribosome-seq data of tomato (BioProject ID number PRJNA514710) and maize (BioProject ID number PRJNA822292). The transcriptome references were downloaded from the Phytozome database (rice v7.0; tomato ITAG4.0; maize RefGen_V4) and the Ensembl Plants database (durum wheat Svevo RefSeq 1.0). The durum wheat reference was optimized following instructions (Yang et al. 2021). For four species, the corresponding adapters were trimmed by Trim Galore v0.6.7. Salmon software (Patro et al. 2017) was used to map the raw reads to transcriptome references and quantify the read counts, followed by calculation of Fragments Per Kilobase of transcript per Million (FPKM) mapped reads (Yang et al. 2021). Translation efficiencies of genes are calculated by dividing the mean FPKM values of polysome-seq/ribosome-seq replicates by the mean FPKM values of RNA-seq replicates. To avoid the bias caused by different isoforms, we only counted the longest isoform of each gene locus, and the genes with mean FPKM values of RNA-seq replicates less than 1 were excluded from further analysis.

### Analysis of the relationship between translation efficiency and iM-related features

Thirty-four iM-related features were extracted according to the iM sequences, including C-tract length, iM length, loop length, middle loop length, longest side loop length, shortest side loop length, sum of two side loops, longest loop length, shortest loop length, A density in iMs, C density in iMs, G density in iMs, U density in iMs, A density in loops, C density in loops, G density in loops, U density in loops, A density in middle loop, C density in middle loop, G density in middle loop, U density in middle loop, A density in longest side loop, C density in longest side loop, G density in longest side loop, U density in longest side loop, A density in shortest side loop, C density in shortest side loop, G density in shortest side loop, U density in shortest side loop, A density in two side loops, C density in two side loops, G density in two side loops, U density in two side loops, and iM strength (iM stability score) predicted by iM-Seeker. Spearman correlations (SPCC) and significance values were calculated by Python scripts. The measurements of mutual information (MI) between TE and each feature were performed by the function mutual_info_regression in the scikit-learn package (Pedregosa et al. 2011). To calculate the significance of MI, we implemented a permutation-based significance test following a strategy similar to that used in previous studies (Goodarzi et al. 2012). Specifically, we randomly shuffled the TE labels 1,000 times and recalculated the MI value for each iM-related feature using the permuted labels, thereby generating a null distribution of MI values. Empirical *P* values were then defined as the proportion of permutations in which the MI exceeded the observed MI calculated from the original data. We used a threshold of *P* < 0.05 as the standard for statistical significance. In addition to the original *U* test, we implemented two complementary approaches. First, we applied Benjamini–Hochberg (FDR) correction to the *P* values obtained from the *U-test* to control for multiple testing. Second, we performed a permutation-based significance test. Specifically, similar to the MI analysis, we randomly shuffled the TE labels 1,000 times and performed the *U* test for each permutation to generate a null distribution of *P* values. Empirical *P* values were then defined as the proportion of permutations in which the *P* value was smaller than the observed *P* value obtained from the original data. We used a threshold of permutation-based *P* < 0.05 to define statistical significance. The important features were selected according to three criteria (permutation-based MI *P* < 0.05; *P* value of Mann-Whitney *U* test < 0.05; permutation-based *U* test *P* < 0.05). All the statistical analyses and significance tests were performed by Python scripts.

### Dual-luciferase reporter assay

Two candidate orthologous gene groups were selected from pre-identified orthologous gene groups in the 1,000 Plants initiative (1KP) (Leebens-Mack et al. 2019). Orthologous gene groups were retained if they contained at least 20 monocot species in 43 monocots (one transcript per species) and at least three monocot transcripts with iM-forming sequences in their 5′UTRs. The group IDs and species abbreviations are consistent with 1KP. The abbreviations of the species are as follows: *Maianthemum sp.* (RCUX), *Paraneurachne muelleri* (XUAB), *Juncus inflexus* (CIEA), and *Curculigo sp.* (YJUG). Sequencing-verified vectors were introduced into *Agrobacterium tumefaciens* GV3101 and transiently expressed in leaves of 3- to 4-week-old *Nicotiana benthamiana* via agroinfiltration. At 48 h post-infiltration, three leaf discs (6 mm diameter) were collected and rapidly frozen in liquid nitrogen, then homogenized in Cell Lysis Buffer (TRANS). After centrifugation at 13,000 rpm for 1 min, the clear supernatant was diluted 20-fold with the same lysis buffer. Luciferase activities were measured with the Dual-Luciferase® Reporter Assay System (Promega) following the manufacturer’s protocol. To quantify the mRNA abundance of Firefly luciferase (F-luc) or Renilla luciferase (R-luc), RNA was isolated with the Plant Total RNA Extraction Kit (GENSTONE BIOTECH). cDNA was synthesized using ToloScript All-in-one RT EasyMix for qPCR (TOLOBIO). Quantitative PCR was performed using 2×Q3 SYBR qPCR Master Mix (TOLOBIO) using QuantStudio 5 Real-Time PCR System (Thermofisher) according to the manufacturer’s protocol. Translation efficiency (TE) and protein level or mRNA abundance normalization were calculated as described (Yang et al. 2021). The significance of the TE difference between RNA pairs was calculated by Student’s *t*-test with four biological replicates. Data were presented as mean values + standard error of mean (SEM).

## Supplementary Material

msag152_Supplementary_Data

## Data Availability

The polysome-seq data of rice (BioProject ID number PRJNA1112739) and wheat (BioProject ID number PRJNA723219) data and ribosome-seq data of tomato (BioProject ID number PRJNA514710) and maize (BioProject ID number PRJNA822292) are available on NCBI. iM-Seeker is available at https://github.com/YANGB1/iM-Seeker. The code used for the remaining statistical tests and analyses is available at https://github.com/YANGB1/Plant-RNA-i-Motif-Landscape.
