# Supplementary material for: RNA i-motif landscapes in plant kingdom and their potential functional roles
Source: Mol Biol Evol. 2026 Jun 20;43(7):msag152. doi: 10.1093/molbev/msag152 (PMC13332401; doi:10.1093/molbev/msag152)
Supplement: msag152_Supplementary_Data [file msag152_supplementary_data.zip › iM-plant_manuscript_MBE_Supplementary_F1.pdf]

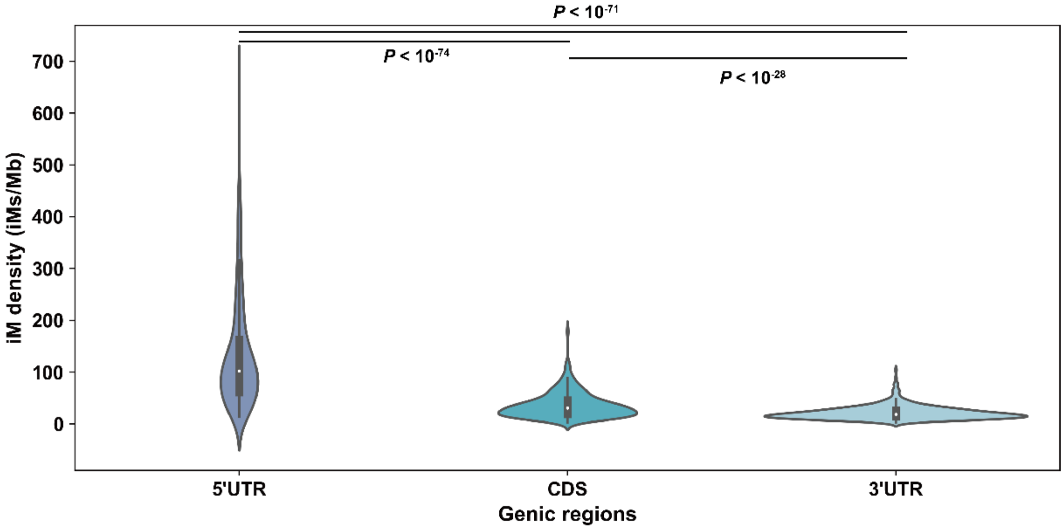

Fig. S1 The comparison of iM densities among genic regions

The comparison of iM densities among three genic regions (5′UTR, CDS and 3′UTR) across 433 land plants with significance tested by paired Students' *t*-test.
