# Supplementary material for: RNA i-motif landscapes in plant kingdom and their potential functional roles
Source: Mol Biol Evol. 2026 Jun 20;43(7):msag152. doi: 10.1093/molbev/msag152 (PMC13332401; doi:10.1093/molbev/msag152)
Supplement: msag152_Supplementary_Data [file msag152_supplementary_data.zip › iM-plant_manuscript_MBE_Supplementary_F3.pdf]

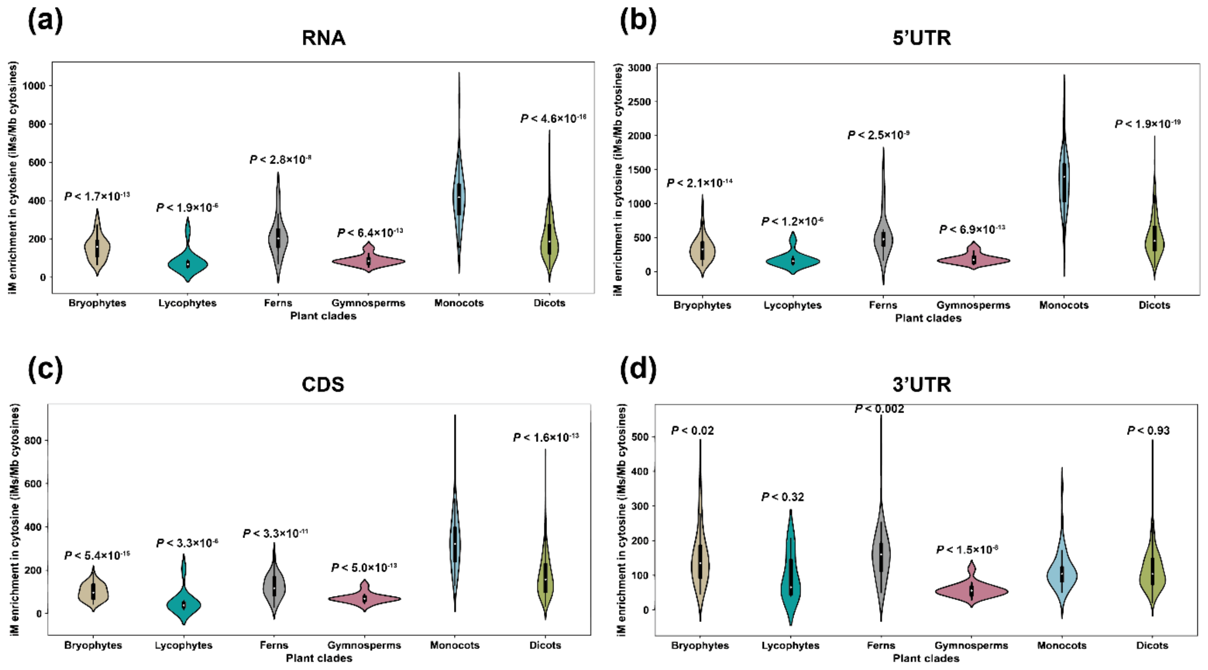

Fig. S3 The iM enrichment across plant transcriptomes

(**a**) The iM enrichment in whole transcriptomes across six clades. (**b**) The iM enrichment in cytosine in 5′UTR regions across six clades. (**c**) The iM enrichment in cytosine in CDS regions across six clades. (**d**) The iM enrichment in cytosine in 3′UTR regions across six clades. Statistical analysis was performed between monocots and other five plant clades with significance tested by Mann-Whitney *u*-test.
