# Supplementary material for: RNA i-motif landscapes in plant kingdom and their potential functional roles
Source: Mol Biol Evol. 2026 Jun 20;43(7):msag152. doi: 10.1093/molbev/msag152 (PMC13332401; doi:10.1093/molbev/msag152)
Supplement: msag152_Supplementary_Data [file msag152_supplementary_data.zip › iM-plant_manuscript_MBE_Supplementary_F4.pdf]

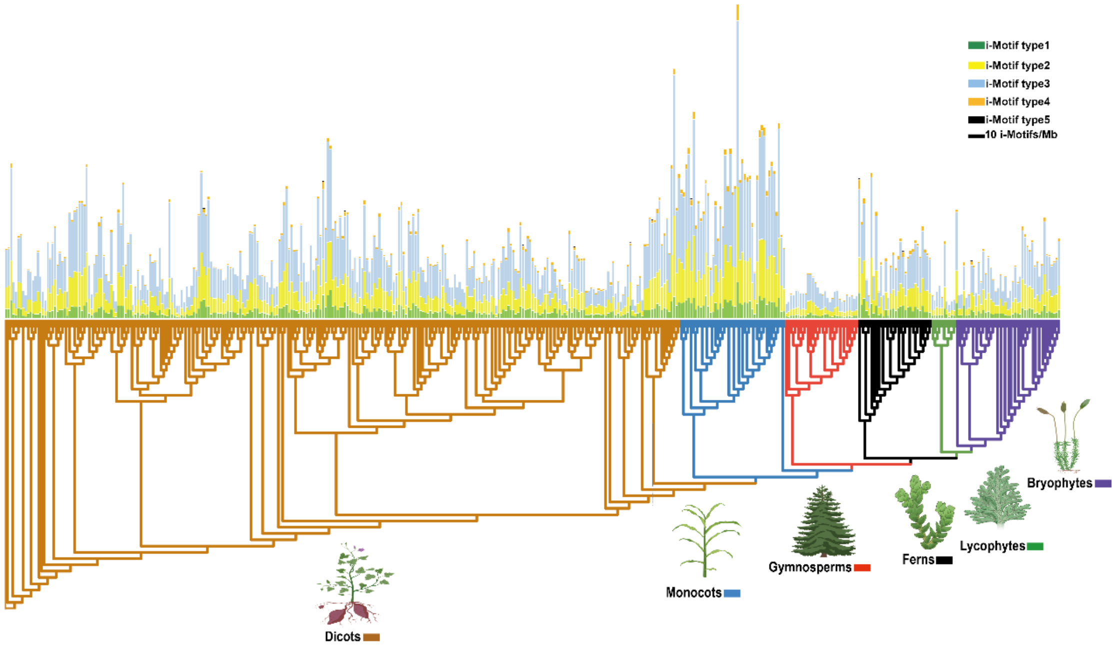

Fig. S4 Transcriptome-wide landscape of RNA i-motif types across the plant kingdom

The landscape of transcriptome-wide i-motifs of different types across 433 land plants. The plants are in six clades. N= 277, 43, 30, 30, 10, 43 for dicots, monocots, gymnosperms, ferns, lycophytes, and bryophytes, respectively. The iMs are divided into 5 categories. Type 1 indicates iMs with three-cytosine C-tracts and longest loop between one and four; Type 2 indicates iMs with three-cytosine C-tracts and longest loop between five and eight; Type 3 indicates iMs with three-cytosine C-tracts and longest loop between nine and twelve; Type 4 indicates iMs with four-cytosine C-tracts; Type 5 indicates iMs with C-tracts length longer than four.
