# Supplementary material for: RNA i-motif landscapes in plant kingdom and their potential functional roles
Source: Mol Biol Evol. 2026 Jun 20;43(7):msag152. doi: 10.1093/molbev/msag152 (PMC13332401; doi:10.1093/molbev/msag152)
Supplement: msag152_Supplementary_Data [file msag152_supplementary_data.zip › iM-plant_manuscript_MBE_Supplementary_F5.pdf]

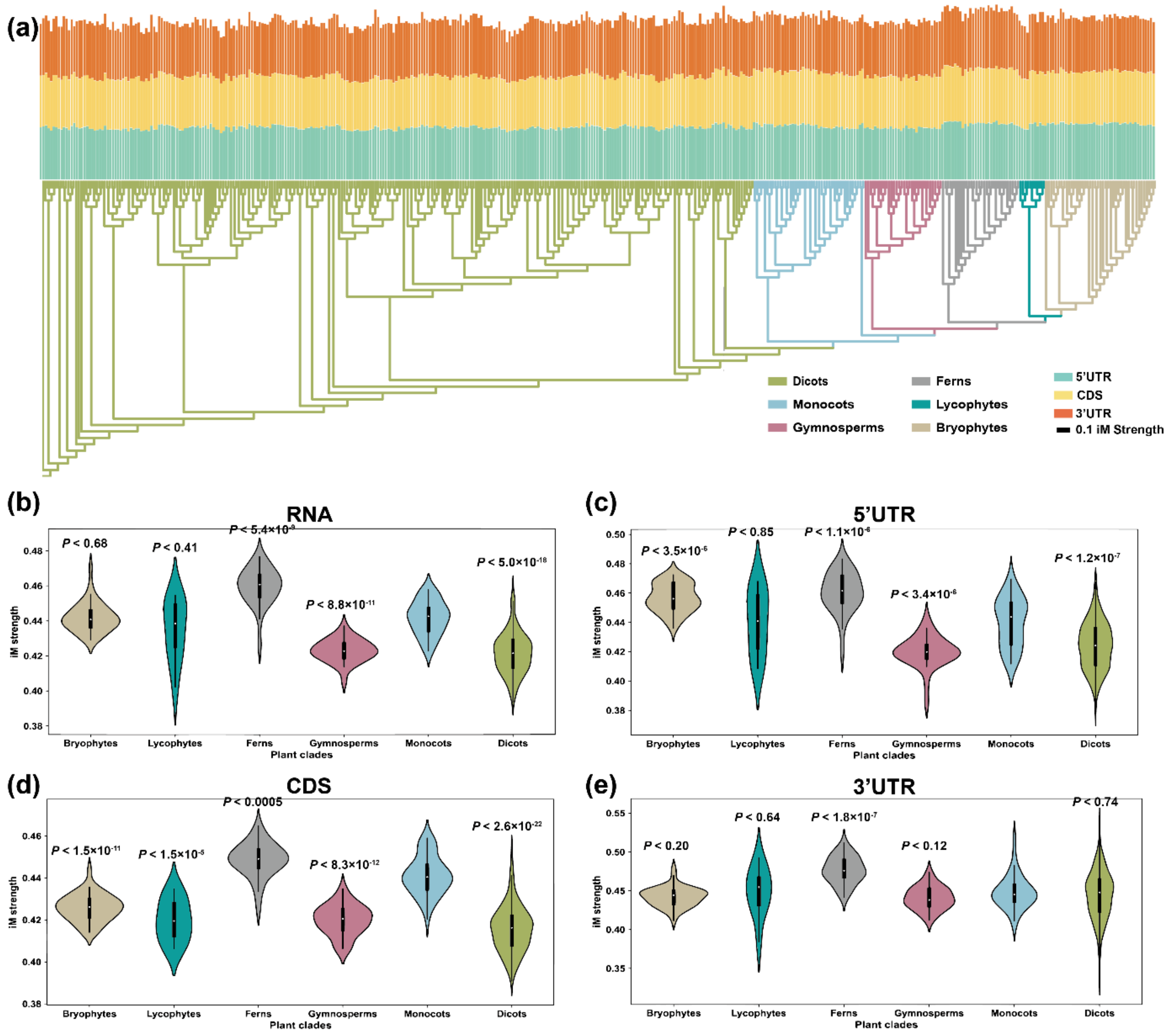

Fig. S5 The transcriptome-wide iM folding strengths in plant kingdom

(**a**) The landscape of transcriptome-wide i-motifs mean folding strength of different genic regions (5′UTR, CDS, and 3′UTR) predicted by iM-Seeker across 433 land plants. The plants are in six clades. n = 277, 43, 30, 30, 10, 43 for dicots, monocots, gymnosperms, ferns, lycophytes, and bryophytes, respectively. (**b**) The distribution of iM strength predicted by iM-Seeker in whole transcriptomes across six clades. (**c**) The distribution of iM strength predicted by iM-Seeker in 5′UTR regions across six clades. (**d**) The distribution of iM strength predicted by iM-Seeker in CDS regions across six clades. (**e**) The distribution of iM strength predicted by iM-Seeker in 3′UTR regions across six clades. Statistical analysis was performed between monocots and other five plant clades with significance tested by Mann-Whitney *u*-test.
