# Supplementary material for: RNA i-motif landscapes in plant kingdom and their potential functional roles
Source: Mol Biol Evol. 2026 Jun 20;43(7):msag152. doi: 10.1093/molbev/msag152 (PMC13332401; doi:10.1093/molbev/msag152)
Supplement: msag152_Supplementary_Data [file msag152_supplementary_data.zip › iM-plant_manuscript_MBE_Supplementary_F6.pdf]

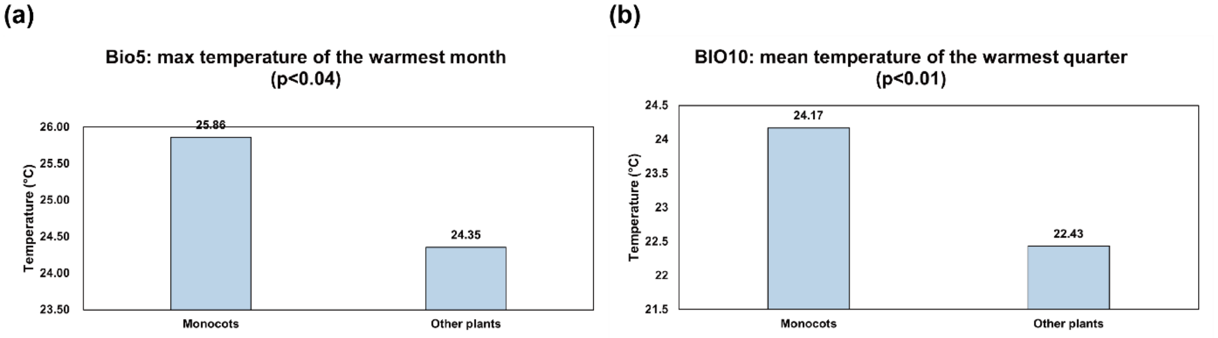

Fig. S6 The comparison of temperature variables between monocots and other plants

The mean value of BIO5 (**a**) and BIO10 (**b**) between monocots and other plants. Statistical analysis was performed between monocots and other plants with significance tested by Mann-Whitney *u*-test.
