# Supplementary material for: RNA i-motif landscapes in plant kingdom and their potential functional roles
Source: Mol Biol Evol. 2026 Jun 20;43(7):msag152. doi: 10.1093/molbev/msag152 (PMC13332401; doi:10.1093/molbev/msag152)
Supplement: msag152_Supplementary_Data [file msag152_supplementary_data.zip › iM-plant_manuscript_MBE_Supplementary_T1.pdf]

Table S1 The percentage of iMs of different types in six plant clades

iM type 1 indicates iMs with three-cytosine C-tracts and longest loop between one and four; iM type 2 indicates iMs with three-cytosine C-tracts and longest loop between five and eight; iM type 3 indicates iMs with three-cytosine C-tracts and longest loop between nine and twelve; iM type 4 indicates iMs with four-cytosine C-tracts; iM type 5 indicates iMs with C-tracts length longer than four.

	iM type1	iM type2	iM type3	iM type4	iM type5
Dicot	11.74%	28.94%	56.47%	2.65%	0.20%
Monocot	10.83%	30.67%	54.97%	3.37%	0.16%
Gymnosperm	9.88%	26.91%	60.19%	2.87%	0.15%
Fern	13.76%	29.44%	51.85%	4.47%	0.48%
Lycophyte	13.07%	27.90%	55.22%	3.56%	0.25%
Bryophyte	11.68%	29.66%	55.11%	3.29%	0.26%
All plants	11.67%	29.29%	55.84%	2.98%	0.22%
