# Supplementary material for: RNA i-motif landscapes in plant kingdom and their potential functional roles
Source: Mol Biol Evol. 2026 Jun 20;43(7):msag152. doi: 10.1093/molbev/msag152 (PMC13332401; doi:10.1093/molbev/msag152)
Supplement: msag152_Supplementary_Data [file msag152_supplementary_data.zip › iM-plant_manuscript_MBE_Supplementary_T2.pdf]

Table S2 The phylogenetic generalized least squares (PGLS) analyses between iM density and environmental variables. The PGLS coefficients (both value and *P*) are included in the table.

	mRNA		5′UTR		CDS		3′UTR	
	Value	*P*	Value	*P*	Value	*P*	Value	*P*
Bio1	1.21	0	5.22	0	0.96	0	0.22	0.04
Bio2	3.05	0	12.51	0	2.68	0	-0.71	0.02
Bio3	0.26	0.02	1.10	0.01	0.18	0.05	0.11	0.06
Bio4	0.003	0.67	0.003	0.87	0.003	0.63	-0.009	0.005
Bio5	1.85	0	7.71	0	1.59	0	0.08	0.49
Bio6	0.61	0.002	2.57	0.0008	0.44	0.009	0.25	0.005
Bio7	0.46	0.03	1.62	0.06	0.57	0.004	-0.33	0.002
Bio8	0.99	0	4.76	0	0.77	0.0001	0.12	0.15
Bio9	0.97	0	4.20	0	0.74	0.0001	0.14	0.09
Bio10	1.82	0	7.73	0	1.51	0	0.18	0.16
Bio11	0.72	0.0001	3.10	0	0.56	0.0005	0.20	0.02
Bio12	-0.005	0.04	-0.03	0.002	-0.005	0.02	0.004	0.0009
Bio13	-0.001	0.92	-0.10	0.16	0.07	0.02	0.02	0.007
Bio14	-0.12	0.03	-0.56	0.01	-0.11	0.04	0.01	0.67
Bio15	0.14	0.003	0.50	0.006	0.12	0.002	-0.003	0.91
Bio16	-0.002	0.73	-0.05	0.09	0.02	0.04	0.007	0.006
Bio17	-0.03	0.04	-0.16	0.01	-0.03	0.07	0.004	0.65
Bio18	0.001	0.82	-0.04	0.35	-0.0003	0.95	0.006	0.04
Bio19	-0.03	0.11	-0.15	0.03	-0.02	0.17	0.01	0.001
