# Supplementary material for: RNA i-motif landscapes in plant kingdom and their potential functional roles
Source: Mol Biol Evol. 2026 Jun 20;43(7):msag152. doi: 10.1093/molbev/msag152 (PMC13332401; doi:10.1093/molbev/msag152)
Supplement: msag152_Supplementary_Data [file msag152_supplementary_data.zip › iM-plant_manuscript_MBE_Supplementary_T3.pdf]

**Table S3** The phylogenetic generalized least squares (PGLS) analyses between iM enrichment and environmental variables. The PGLS coefficients (both value and *P*) are included in the table.

| | mRNA | | 5′UTR | | CDS | | 3′UTR | |
|---|---|---|---|---|---|---|---|---|
| | Value | *P* | Value | *P* | Value | *P* | Value | *P* |
| Bio1 | 5.71 | 0 | 19.79 | 0 | 4.50 | 0 | 1.58 | 0.003 |
| Bio2 | 14.31 | 0 | 47.13 | 0 | 12.43 | 0 | -2.56 | 0.08 |
| Bio3 | 1.24 | 0.01 | 4.23 | 0.008 | 0.88 | 0.04 | 0.65 | 0.02 |
| Bio4 | 0.02 | 0.56 | 0.03 | 0.78 | 0.02 | 0.52 | -0.04 | 0.01 |
| Bio5 | 8.66 | 0 | 29.07 | 0 | 7.35 | 0 | 1.13 | 0.06 |
| Bio6 | 2.84 | 0.001 | 9.85 | 0.0005 | 2.04 | 0.006 | 1.48 | 0.0009 |
| Bio7 | 2.26 | 0.02 | 6.42 | 0.04 | 2.75 | 0.002 | -1.35 | 0.008 |
| Bio8 | 4.78 | 0 | 18.02 | 0 | 3.67 | 0 | 1.02 | 0.02 |
| Bio9 | 4.52 | 0 | 15.90 | 0 | 3.44 | 0 | 0.94 | 0.02 |
| Bio10 | 8.61 | 0 | 29.44 | 0 | 7.08 | 0 | 1.66 | 0.01 |
| Bio11 | 3.42 | 0 | 11.83 | 0 | 2.59 | 0.0003 | 1.26 | 0.003 |
| Bio12 | -0.02 | 0.04 | -0.11 | 0.002 | -0.02 | 0.02 | 0.02 | 0.002 |
| Bio13 | 0.002 | 0.97 | -0.33 | 0.22 | 0.33 | 0.01 | 0.09 | 0.004 |
| Bio14 | -0.55 | 0.03 | -2.16 | 0.007 | -0.54 | 0.03 | 0.02 | 0.90 |
| Bio15 | 0.66 | 0.001 | 1.93 | 0.003 | 0.58 | 0.0009 | 0.06 | 0.67 |
| Bio16 | -0.006 | 0.80 | -0.16 | 0.12 | 0.11 | 0.02 | 0.03 | 0.005 |
| Bio17 | -0.15 | 0.03 | -0.62 | 0.009 | -0.16 | 0.06 | 0.009 | 0.84 |
| Bio18 | 0.01 | 0.64 | -0.12 | 0.42 | 0.004 | 0.86 | 0.04 | 0.01 |
| Bio19 | -0.13 | 0.11 | -0.54 | 0.03 | -0.10 | 0.16 | 0.05 | 0.007 |
