# Supplementary material for: RNA i-motif landscapes in plant kingdom and their potential functional roles
Source: Mol Biol Evol. 2026 Jun 20;43(7):msag152. doi: 10.1093/molbev/msag152 (PMC13332401; doi:10.1093/molbev/msag152)
Supplement: msag152_Supplementary_Data [file msag152_supplementary_data.zip › iM-plant_manuscript_MBE_Supplementary_T4.pdf]

Table S4 The phylogenetic generalized least squares (PGLS) analyses between C density and environmental variables. The PGLS coefficients (both value and *P*) are included in the table.

	mRNA		5′UTR		CDS		3′UTR	
	Value	*P*	Value	*P*	Value	*P*	Value	*P*
Bio1	-0.00007	0.50	0.0005	0.001	-0.0001	0.24	-0.0003	0.04
Bio2	-0.0005	0.054	0.001	0.01	-0.0006	0.01	-0.001	1e-04
Bio3	-0.00002	0.67	0.0001	0.27	-0.00004	0.42	-0.00001	0.88
Bio4	-0.0000005	0.07	0.000001	0.81	-0.000006	0.03	-0.00001	3e-04
Bio5	-0.0001	0.25	0.0008	0	-0.0002	0.11	-0.0007	0
Bio6	0.00004	0.64	0.0002	0.07	0.00001	0.88	-0.00002	0.87
Bio7	-0.0002	0.03	0.0002	0.29	-0.0003	0.005	-0.0005	0
Bio8	-0.0002	0.01	0.0004	0.002	-0.0003	0.002	-0.0003	0.006
Bio9	0.00003	0.72	0.0005	0.002	-0.000009	0.93	-0.0002	0.09
Bio10	-0.0002	0.10	0.0007	3e-04	-0.0003	0.03	-0.0006	1e-04
Bio11	0.000008	0.92	0.0003	0.02	-0.00002	0.77	-0.00006	0.53
Bio12	0.00000005	0.96	-0.000003	0.09	-0.00000001	0.99	0.000003	0.02
Bio13	-0.000005	0.39	-0.00002	0.14	-0.00003	0.052	0.000006	0.41
Bio14	0.00001	0.67	-0.00002	0.59	0.00002	0.56	0.00005	0.19
Bio15	-0.00003	0.14	0.00002	0.51	-0.00004	0.08	-0.00005	0.12
Bio16	-0.000001	0.58	-0.000008	0.11	-0.00001	0.07	0.000004	0.19
Bio17	0.000003	0.70	-0.000005	0.66	0.000002	0.86	9.4e-06	0.37
Bio18	-0.000005	0.08	-0.000007	0.36	-0.000006	0.03	-0.000004	0.30
Bio19	-0.000003	0.73	-0.00002	0.16	0.000001	0.85	0.00002	0
